# GABAergic Signaling as Therapeutic Target for Autism Spectrum Disorders

**DOI:** 10.3389/fped.2014.00070

**Published:** 2014-07-08

**Authors:** Giada Cellot, Enrico Cherubini

**Affiliations:** ^1^Department of Neuroscience, Scuola Internazionale Superiore di Studi Avanzati, Trieste, Italy; ^2^European Brain Research Institute, Rome, Italy

**Keywords:** autism spectrum disorders, GABA receptors, bumetanide, neuro-developmental disorders, excitatory inhibitory balance

## Abstract

γ-Aminobutyric acid (GABA), the main inhibitory neurotransmitter in the adult brain, early in postnatal life exerts a depolarizing and excitatory action. This depends on accumulation of chloride inside the cell via the cation–chloride importer NKCC1, being the expression of the chloride exporter KCC2 very low at birth. The developmentally regulated expression of KCC2 results in extrusion of chloride with age and a shift of GABA from the depolarizing to the hyperpolarizing direction. The depolarizing action of GABA leads to intracellular calcium rise through voltage-dependent calcium channels and/or *N*-methyl-d-aspartate receptors. GABA-mediated calcium signals regulate a variety of developmental processes from cell proliferation migration, differentiation, synapse maturation, and neuronal wiring. Therefore, it is not surprising that some forms of neuro-developmental disorders such as autism spectrum disorders (ASDs) are associated with alterations of GABAergic signaling and impairment of the excitatory/inhibitory balance in selective neuronal circuits. In this review, we will discuss how changes of GABA_A_-mediated neurotransmission affect several forms of ASDs including the Fragile X, the Angelman, and Rett syndromes. Then, we will describe various animal models of ASDs with GABAergic dysfunctions, highlighting their behavioral deficits and the possibility to rescue them by targeting selective components of the GABAergic synapse. In particular, we will discuss how in some cases, reverting the polarity of GABA responses from the depolarizing to the hyperpolarizing direction with the diuretic bumetanide, a selective blocker of NKCC1, may have beneficial effects on ASDs, thus opening new therapeutic perspectives for the treatment of these devastating disorders.

Autism comprises a heterogeneous group of neuro-developmental disorders known as autism spectrum disorders (ASDs) characterized by deficits in verbal and non-verbal communication, social interaction, restricted interests, and stereotyped behavior (1). The incidence of ASDs (20–60/10000 children) has dramatically increased over the past decades because of the improvement of diagnostic criteria and increased attention of medical community (2). Complications arising from later age pregnancies and from excessive exposure of fetuses with a genetic vulnerable background to environmental factors (i.e., toxic agents) may also contribute to the higher incidence of these disorders in recent years.

In spite different etiologies, ASDs share overlapping symptoms, indicating common deficits in some neuro-developmental pathways. One of these involves the γ-aminobutyric acid (GABA)_A_-mediated neurotransmission, known to play a crucial role in synaptic tuning and neuronal wiring in late pre and early postnatal days (3). Studies from animal models of ASDs indicate that a dysfunction in GABAergic signaling within particular neuronal circuits may account for most of the clinical symptoms found in autistic patients. The high co-morbidity of ASDs with epilepsy (30% of cases) further confirms this issue (4).

γ-Aminobutyric acid is the main inhibitory neurotransmitter in the adult mammalian brain. It inhibits neuronal firing by activating two different classes of receptors, GABA_A_ and GABA_B_. GABA_A_ receptors are integral ion channels while GABA_B_ receptors are coupled to ion channels via guanine nucleotide-binding proteins and second messengers. The opening of GABA_A_ receptors causes a net influx of chloride with consequent membrane hyperpolarization and reduction of cell firing. However, in particular conditions and during brain maturation the intracellular chloride concentration [Cl^−^]_i_ rises in such a way that the opening of anion channels by GABA produces a chloride efflux and a membrane depolarization that through the activation of a persistent non-inactivating sodium conductance (5) may reach the threshold for action potential generation (6, 7). Generally, low [Cl^−^]_i_ facilitates GABA-mediated inhibition, whereas high [Cl^−^]_i_ facilitates GABA-mediated excitation. The mechanisms underlying chloride accumulation inside immature neurons, start to be unveiled with a different efficacy of chloride co-transporters such as NKCC1 and KCC2, which import and export chloride, respectively. Before and immediately after birth, chloride accumulates inside the cell due to a reduced expression of the cation–chloride exporter KCC2. Later in development, the intracellular chloride concentration decreases thanks to the up-regulation of KCC2 (8–10). GABA-induced membrane depolarization facilitates calcium entry via voltage-dependent calcium channels and *N*-methyl-d-aspartate (NMDA) receptors. Calcium rise leads to the activation of second messengers involved in a variety of developmental processes from cell migration and differentiation to synaptogenesis and circuit formation (11).

How GABA orchestrates these processes has been extensively reported (3, 12–14). GABAergic signals operate with multiple modalities at different developmental stages before glutamatergic ones (15). At the beginning, GABA works as a trophic factor, modulating neuronal migration and maturation (16). GABA receptors are expressed in neuronal progenitors before the establishment of synaptic contacts (17). At this stage, the receptors work as sensors for GABA present in the extracellular space after its release in a calcium- and SNARE-independent way from growth cones and astrocytes (18). The absence of an efficient uptake system enables this neurotransmitter to accumulate in the extracellular space and to reach a concentration sufficient to exert its depolarizing and excitatory effects on distal neurons. Blocking the depolarizing action *in utero* heavily affects migration and circuit formation (11, 19, 20).

At later developmental stages, when synapses are formed, the release of GABA and glutamate, generate a primitive form of network-driven oscillatory events known as giant depolarizing potentials (GDPs). GDPs are characterized by recurrent membrane depolarizations (lasting several hundred of milliseconds) that give rise to bursts of action potentials, separated by quiescent periods. This network activity thought to be the *in vitro* counterpart of “sharp waves” recorded in pups during immobility periods, sleep, and feeding (21), is reminiscent of the “trace discontinue” first described by Dreyfus-Brisac in the electroencephalogram of immature babies and characterized by intermittent bursts separated by periods of virtually complete suppression of activity (22).

In analogy with the synchronized activity generated in the disinhibited hippocampus by GABA_A_ receptor antagonists (23), GDPs emerge when a sufficient number of cells fire and the excitability of the network attains a certain threshold within a restricted temporal window (24). Although the entire hippocampus possesses the capacity to generate GDPs, for its extensive glutamatergic connections via recurrent collaterals, the CA3 area is particularly well equipped to generate synchronized activity. Furthermore, this area is able to initiate, upon membrane depolarization, intrinsic bursts which, by virtue of their spontaneous discharges and large spike output can drive other neurons to fire (25, 26). Burst firing is facilitated by a persistent slow sodium current (27) and by a tonic GABA_A_-mediated conductance generated by the activation of extrasynaptic GABA_A_ receptors by “ambient” GABA whose depolarizing action would bring the membrane to the voltage window for activation of voltage-dependent sodium and calcium channels (28). Intrinsic bursting activity is boosted by the low expression of Kv7.2 and Kv7.3 channels responsible for the non-inactivating, low-threshold M current (*I*_M_), which in adulthood controls spike after-depolarization and burst generation (29). The low density of *I*_M_ at birth contributes to produce intrinsic bursts that, in comparison with those observed in adults, are more robust, last longer and recur more regularly (25). GDPs-associated calcium transients act as coincident detector signals for enhancing synaptic efficacy at emerging GABAergic (30) and glutamatergic synapses (31). Therefore, this early synchronized activity is fundamental for synaptic wiring and refinement of local neuronal circuits according to the Hebbian rule that “neurons that fire together wire together.”

γ-Aminobutyric acid is released from GABAergic interneurons that constitute a very heterogeneous group of cells, differentially classified according to their morphology, biophysical properties, molecular expression profile, and connectivity (32). These cells, mainly derived from the medial and caudal ganglionic eminences, undergo their final mitosis in these regions prior to their tangential migration into the cortical plate. The migration process, supposed to be calcium dependent, is regulated by a sequence of well-orchestrated processes involving guidance cues, neurotransmitter receptors (NMDA, GABA_A_ receptors) and voltage-dependent calcium channels (33, 34).

GABAergic interneurons not only exert a powerful control on network excitability but, in spite of their relatively low number (10–15% of the entire neuronal population), are able to synchronize a large number of principal cells giving rise to coherent oscillations, which support different behavioral states of the animals and high cognitive tasks (35).

Altogether, these observations point to GABA as one of the major players in the early assembly and formation of neuronal circuits in the developing brain. Therefore, it is not surprising that dysfunctions of GABAergic circuits have been implicated in various neuro-developmental and psychiatric disorders such as schizophrenia, autism, and epilepsy.

## GABAergic Dysfunctions in the Brain of ASD Patients

The high frequency of epileptiform activity and the altered brain rhythms detected in the EEG of ASDs patients suggest a dysfunction of GABAergic transmission and an imbalance between excitation and inhibition (E/I) in local circuits involved in sensory, mnemonic, social, and emotional processes. However, as summarized in Table 1, more direct evidence in favor of a GABAergic dysfunction in ASDs derives from:

- Genetic observations
- *In vitro* analysis of post-mortem brain tissues
- *In vivo* studies on patients affected mainly by idiopathic forms of ASDs.

**Table 1:** **Alterations of GABAergic signaling in patients with idiopathic forms of autism and Rett syndrome**.

Clinical phenotype	Alterations of GABAergic signaling	Age (years)	Reference
**GENETIC OBSERVATIONS**			
Idiopathic autism	Linkage disequilibrium in *GABRB3*	7.6 ± 6.2	(36, 37)
Idiopathic autism	Altered gene expression in interneurons	Not specified	(38)
***IN VITRO* ANALYSIS OF POST-MORTEM BRAIN TISSUES**			
Idiopathic autism	Reduction in the density of GABA_A_, GABA_B_ receptors and benzodiazepine binding sites in the anterior cingulate cortex	19/43	(39)
Idiopathic autism	Reduction in the density of GABA_B_ receptors cingulate cortex and fusiform gyrus	19/43	(40)
Idiopathic autism	Reduction in 3[H]flunitrazepam labeled benzodiazepines binding sites in the hippocampus	16/22	(41)
Idiopathic autism	Decreased number of GABAergic Purkinje cells in cerebellum	13/54	(42)
Idiopathic autism	Reduced level of GAD65 and GAD67 in Purkinje cells of cerebellar and parietal cortices	19/30	(43)
Idiopathic autism	Decreased GAD67 mRNA levels in cerebellar Purkinje cells	16/30	(44)
Idiopathic autism	Decreased GAD65 mRNA levels in cerebellar dentate nuclei	16/30	(45)
Idiopathic autism	Increased expression of GABAergic interneurons expressing calcium-binding proteins in the hippocampus	13/63	(46)
Rett syndrome	Disruption in the inhibitory architecture of the cell mini-columns	14.4 ± 4	(47)
***IN VIVO* STUDIES**			
Idiopathic autism	Reduction of GABA concentration in the frontal lobe	2/12	(48)
Idiopathic autism	Reduced GABA levels in the perisylvian region of the left hemisphere	12.4 ± 5.2	(49)
Idiopathic autism	Reduced expression of GABA_A_ receptors in the superior and medial frontal cortex	7.3 ± 3.5	(50)
Rett syndrome	Reduced GABA_A_ receptor density in fronto-temporal cortex	27/41	(51)
Idiopathic autism	Significant reduction of α5 GABA_A_ receptor subunits in limbic areas	34/43	(52)
Rett syndrome	Reduced KCC2/NKCC1 ratio in the cerebrospinal fluid	0/19	(53)

*Numbers refer to mean (usually ± SEM) or range values*.

It is clear from the Table 1 that most of cases are from juvenile and adult patients. This can be attributed to difficulties in obtained post-mortem material from young children and to perform complex *in vivo* examinations such as positron emission tomography (PET) and single photon emission computed tomography (SPECT) in children of pediatric age. However, we cannot exclude that the same alterations are already present at early stages of development.

### Genetic observations

The involvement of GABA_A_ receptors in ASDs was provided by genetic studies that have revealed submicroscopic abnormalities known as “copy-number variations” in chromosomal loci 15q11–q13, which contains a number of genes encoding for GABA_A_ receptor subunits (54). These loci can be affected either directly by single point mutations or indirectly by epigenetic factors. Potential gene targets include *GABRB3, GABRA5*, and *GABRG3*, encoding for β3, α5, and γ3 subunits containing GABA_A_ receptors, respectively (36, 37). Systematic changes in GABA_A_ receptor subunit expression were found in the superior frontal cortex, parietal cortex, and cerebellum of autistic subjects. In addition, autism-related genes have been found to be expressed mainly in GABAergic interneurons (38).

### *In vitro* analysis of post-mortem brain tissues

Post-mortem analysis on brain tissues from ASD patients as well as genetic and *in vivo* studies have largely contributed to unveil the impact of GABAergic signaling in these disorders. Thus, as compared to controls, a significant reduction in the density of GABA_A_, GABA_B_ receptors, and benzodiazepine binding sites was detected in the supra and infragranular layers of the anterior cingulate cortex (known to participate in a variety of processes including socio-emotional behavior and other associative functions via prefrontal cortex connectivity) in brain samples from autistic subjects (39, 40). A reduction of ^3^[H]muscimol labeled GABA_A_ receptors and 3[H]flunitrazepam labeled benzodiazepines binding sites was found also in the hippocampus (41, 55). Neuropathological studies from the cerebellum of individuals with ASDs have demonstrated that GABAergic Purkinje cells are particularly vulnerable since their number appears considerably reduced respect to controls (42, 56, 57). This effect was found to be associated with reduced levels of mRNA encoding for glutamic acid decarboxylase (GAD) 65 and 67, rate limiting enzymes responsible for the conversion of glutamate to GABA (43–45, 58). In contrast with Purkinje cells, enhanced levels of mRNA for GAD67 were found in stellate cells, a subtype of interneuron innervating Purkinje cells (43). Interestingly, pathological studies of brains of individual affected by autism demonstrated an increased expression of GABAergic interneurons expressing calcium-binding proteins such as calbindin-, calretinin-, and parvalbumin in the hippocampal formation (46). Since these interneurons are capable of buffering calcium, the intracellular messenger that controls several transduction pathways, an alteration of calcium signaling may have dramatic consequences on neuronal functions and dynamics.

Abnormalities in micro-columnar organization of prefrontal cortex in brain tissues from autistic individuals, including two with Angelman syndrome, have been also detected (59, 60). The narrower size of mini-columns in autistic patients respect to controls may reflect defects in GABAergic fibers within and between cortical mini-columns due to reductions in the neuropil, which separates adjacent mini-columns (47). This may alter local connectivity and lateral inhibition.

### *In vivo* studies on patients affected mainly by idiopathic forms of ASDs

*In vivo* studies from ASD patients are rather limited due to the difficulty of measuring GABAergic function *in vivo*. Using proton magnetic resonance spectroscopy, Harada et al. (48) reported a reduction of GABA concentration in the frontal lobe of children with ASDs respect to controls. However, in this study ASD patients were sedated with triclofos a compound that, by potentiating GABA action, may have biased the results. Using the same technique, Rojas et al. (49) have reported reduced GABA levels in the perisylvian region of the left hemisphere of autistic patients, further supporting the involvement of GABAergic neurotransmission in ASDs. Using SPECT and ^123^I-iomazenil, a selective GABA_A_-benzodiazepine ligand, a reduced expression of GABA_A_ receptors in the superior and medial frontal cortex of autistic patients was detected (50). Using the same technique, a reduced GABA_A_ receptor density was observed in three adult females affected by the Rett syndrome (51). In addition, a pilot study using PET and [^11^C]Ro15-4513 to measure the expression levels of α5 GABA_A_ receptor subunits (localized mainly to extrasynaptic regions where they mediate tonic inhibition), has demonstrated a significant reduction of these subunits in limbic areas of ASD patients respect to controls (52).

Indirect evidence for deficits of GABAergic transmission in ASDs was provided by a recent magneto-encephalographic study that revealed impaired gamma-band activity in selective brain areas of adults with autism during perceptual visual processing (61). Gamma-band oscillations known to critically depend on negative feedback inhibition of principal cells by GABAergic interneurons, including parvalbumin-positive ones (62), are crucial for integrating multisensory information into a coherent representation (63). Although no data from young ASD patients are available, the possibility that such impairment may result from aberrant pre and perinatal development cannot be excluded.

Although informative, these studies referred mainly to idiopathic forms of ASDs and failed to address whether particular types of autistic syndromes, including monogenic ones were similarly affected. In this perspective, it is worth noting that a significant reduction of the cation–chloride importer KCC2 in the cerebrospinal fluid of Rett syndrome patients as compared to controls, with a consequent reduction in the KCC2/NKCC1 ratio was found (53). In accord with the notion that the methyl-CpG-binding protein 2 (MeCP2), encoded by the X-linked *Mecp2* gene, is highly expressed in GABAergic interneurons where it regulates their function these data suggest that a GABA dysfunction underlies the pathophysiology of the Rett syndrome.

Altogether, these findings indicate that alterations of GABAergic signaling in selective microcircuits of well-defined brain areas exert a fundamental role in the pathogenesis of ASDs.

## GABAergic Dysfunctions in Animal Models of ASDs

**Figure 1:**
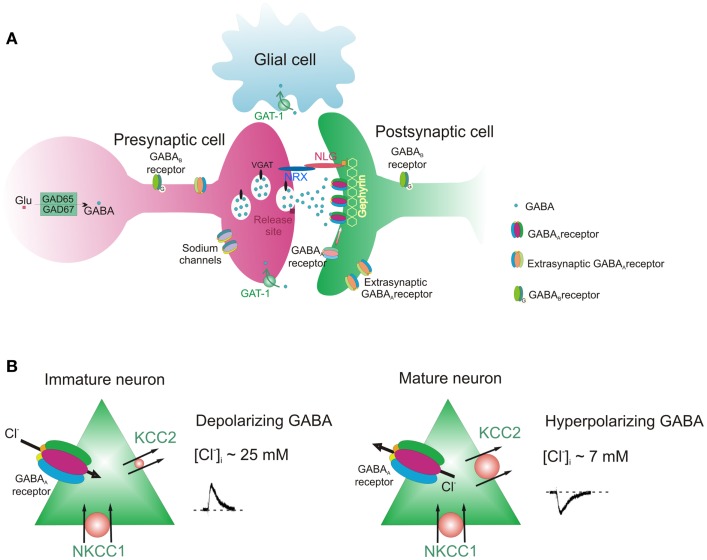
**GABAergic synapse**. **(A)** Pre and post-synaptic sites of the synapse with different components whose functions can be altered in ASDs. **(B)** Direction of GABA action (depolarizing and hyperpolarizing) in immature and mature neurons.

Over the last decade several animal models of ASDs have been generated, in order to identify the molecular and cellular mechanisms underlying these disorders and to develop new therapeutic tools. To this aim, genetic defects detected in patients with ASDs have been introduced in the genome of mice. Animal models have been produced not only by manipulating candidate genes but also environmental factors or drugs such as valproic acid (VPA), an antiepileptic known to be a risk factor for autism in offspring of mothers treated during pregnancy with this drug (64). In accordance with human observations, animal studies have revealed important dysfunctions in GABAergic signaling occurring at different locations of the GABAergic synapse (Figure 1).

Table 2 summarizes alterations of GABAergic transmission observed in the most commonly used animal models of ASDs.

**Table 2:** **Alterations of GABAergic signaling in animal models of ASDs (E, embryonic day; P, postnatal day)**.

Animal models	Alterations of GABAergic signaling	Age	Reference
Fmr1 KO *(X Fragile)*	Reduced GABA release in the amygdala	P20–30	(65)
	Reduced number of parvalbumin-positive interneurons	Adult	(66)
	Altered E/I balance	P14–P30	(67)
	Decreased expression of GAD67 and GABA_A_ receptor subunits	Adult	(68)
	Down regulation of GABA_A_-mediated tonic inhibition	Adult	(69)
	Persistent depolarizing effect of GABA in juvenile animals	E20–P30	(70)
MECP2 KO (Rett syndrome)	Altered E/I balance	P14–P35	(71)
	Depressed GABAergic synaptic transmission	P7	(72)
		P14–P28	(73)
		Adult	(74)
	Reduced expression of GAD65 and GAD 67	Adult	(75)
Ube3a-deficient mouse (Angelman syndrome)	Reduced GABA_A_-mediated tonic inhibition	P25–P28 or adult	(76)
GABRB3 KO (Angelman syndrome)	Decreased expression of GABA_A_ receptors	Adult	(77)
*Scn1a*^+/−^mice (Dravet’s syndrome)	Altered E/I balance	P21–P30	(78)
NL3^R451C^KI	Increased GABAergic neurotransmission; increased VGAT and gephyrin expression	P13–P16	(79)
	Increased frequency of GDPs	P4–P35	(14)
	Circuit specific changes in GABAergic signaling in the hippocampus and in the cortex	P21–P35	(80)
		P9–P15	(81)
	Decreased number of PV^+^ interneurons	P21–P35	(82)
En2 KO	Reduction of GABAergic markers during development; reduced number of GABAergic interneurons	Adult	(83)
VPA model	Decreased GABA_A_-mediated neurotransmission	P23–P45	(84)
	Persistent depolarizing effect of GABA in juvenile animals	E20–P30	(70)

The *Fragile X* syndrome is a monogenetic disorder caused by mutations of the *FMR1* gene, located in chromosome X (Xq27.3) and encoding for the *Fragile X* mental retardation protein (FMRP), which is involved in the translation of a variety of mRNAs. Gene mutations do not allow the expression of the FMRP protein, determining a phenotype characterized by intellectual disabilities associated with language deficits, hyperactivity, autistic behavior, and seizures (85). Mice lacking the *FMR1* gene (Fmr1 KO mice) show an increased network excitability associated with an E/I imbalance in cortical circuits involving specific types of interneurons (65, 67, 86, 87). Thus, in the somatosensory cortex, the reduced activity of parvalbumin-positive cells (66) may contribute to enhance cell excitability and to affect gamma frequency oscillations, thought to be involved in high cognitive functions (86). The enhanced coupling of group I metabotropic glutamate receptors (mGluR) signaling and cannabinoid receptors mobilization may further enhance suppression of inhibition (88). Other factors contributing to enhance cell excitability include: (i) a reduced expression of GABA_A_ receptors subunits in the cortex. This may represent an evolutionary conserved hallmark of the *Fragile X* syndrome since similar reduction has been detected in the *Fragile X* fruit fly model (89). (ii) A down regulation of mRNA encoding for GAD65 and GAD67 in presynaptic terminals of the cortex and cerebellum; (iii) a reduction of mRNA encoding for gephyrin, the scaffold molecule responsible for glycine and GABA_A_ receptor clustering and stabilization at post-synaptic sites; (iv) reduced phasic and tonic GABA_A_-mediated conductances in the amygdale associated with a down regulation of GABA_A_ receptor subunits (65, 68, 89); (v) a down regulation of GABA_A_-mediated tonic inhibition associated with a reduced expression of α5 and δ GABA_A_ receptors subunits in subicular neurons (69). In contrast, electrophysiological recordings from the striatum of Fmr1 KO mice have revealed a selective increase in basal inhibitory neurotransmission, caused by the enhanced probability of GABA release (90). Overall these data suggest that in this animal model modifications of the GABAergic function are region-specific. Interestingly, a recent study from the hippocampus of mice carrying the *Fragile X* mutation, has revealed the loss of oxytocin-mediated GABA_A_-mediated inhibition during the delivery process (70). In rats, oxytocin, the maternal hormone involved in delivery, has been shown to cause an abrupt shift of GABA from the depolarizing to the hyperpolarizing direction, thus exerting a neuroprotective and analgesic action on newborns (91). Unlike wild-type animals, the depolarizing action of GABA, apparently caused by the reduced expression of KCC2 with consequent high [Cl^−^]_i_, persists in juvenile life. The increased excitatory drive to principal cells boosts network activity in the hippocampus, suggesting that an altered E/I balance may be caused not only by a decreased GABA_A_-mediated inhibition but also by an enhanced GABA_A_-mediated excitation (70).

Another neuro-developmental disorder showing high co-morbidity with autism is the *Rett syndrome*. This is caused by mutations in the X-linked *Mecp2* gene that codes for the transcriptional factor MeCP2, highly expressed in GABAergic neurons (92). This disorder affects mainly girls who develop normally until the age of 6/18 months. Later, they develop cognitive deficits, loss of speech, motor abnormalities, respiratory dysrhythmias, stereotyped behavior, and seizures leading sometimes to premature death (93). Mice lacking MeCP2 or engineered to express an allele mimicking some mutation present in the *Rett syndrome* exhibit neurological symptoms reminiscent of those found in patients affected by the *Rett syndrome*. Similarly to Fmr1 KO mice, these animals exhibit alterations of the E/I balance (71), associated with a decrease in somatic GABA content and low levels of mRNA encoding for GAD65 and GAD67 in cortical and striatal neurons (75). While in the thalamus, the *Mecp2* gene appears to differentially regulate the development of GABAergic synapses in excitatory and inhibitory neurons (94), in the brain stem, the E/I imbalance is due to the depression of GABAergic transmission originating at both pre- and post-synaptic levels (72). A reduced GABAergic inhibition is present also in the locus coeruleus (73). In the hippocampus of *Mecp2* KO mice, a significant reduction in the quantal size of miniature inhibitory post-synaptic currents (mIPSCs) has been detected. This would account for the impairment of long-term potentiation (LTP) induced at CA3–CA1 synapses by theta burst stimulation (74).

An E/I imbalance has been found also in individuals with *Tuberous sclerosis*, a genetic multisystem disorder characterized by wide spread hamartomas in several organs, including brain, heart, skin, eyes, kidney, lung, and liver (95). Tuberous sclerosis patients exhibit a variety of neurological disorders including mental retardation, autism-like disorders, and epilepsy. The affected genes are *Tsc1* and *Tsc2* encoding hamartin and tuberin, respectively. The hamartin–tuberin complex inhibits the mammalian target of rapamycin pathway that controls cell growth and proliferation (95). Immunocytochemical and western blots experiments have demonstrated that this disorder is associated with a decrease of α1 GABA_A_ receptor subunits and reduced and enhanced levels of KCC2 and NKCC1, respectively, in tubers. Changes in the expression of KCC2 and NKCC1 account for the excitatory action of GABA revealed with patch clamp in slices from *Tuberous sclerosis* tubers (96). However, electrophysiological data are still preliminary and should be taken with caution since they refer only to experiments from cortical slices obtained from tubers of a single patient. The *Angelman* or the closely related *Prader–Willi* syndromes (depending from which parent the deletion has been inherited), characterized by mental retardation, autistic behavior, and seizures, are determined by the loss of function of the *Ube3a* gene encoding for the a ubiquitin E3 ligase or *Gabrb3, Gabra5*, and *Gabrg3* genes, encoding, respectively for β3, α5, and γ3 GABA_A_ receptor subunits localized in the same chromosome region 15q11–q13. Ube3a-deficient mice exhibit a reduced tonic GABA-mediated inhibition in the cerebellum, caused by a GAT-1 dependent decrease of GABA concentration in the extracellular space (76). Moreover, β3-deficient mice, which have a phenotype similar to some forms of *Angelman* syndrome, exhibit a reduced expression of GABA_A_ receptors in selective brain regions as determined by quantitative autoradiography (77).

The *Dravet’s* syndrome is caused by a haploinsufficiency of the *SCN1A* gene encoding for voltage-gated sodium channel Na_V_1.1. Children affected by this disorder show intractable seizures, cognitive deficit, and autism spectrum behaviors. GABA plays a pivotal role also in this disorder as demonstrated by the reduced activity of Na_V_1.1 channels in forebrain GABAergic interneurons of *Scn1a*^+/−^ mice. These animals exhibited an E/I imbalance resulting from a decreased frequency of spontaneous inhibitory post-synaptic currents and an increased frequency of spontaneous excitatory post-synaptic currents in the hippocampus and prefrontal cortex. The impairment of GABAergic neurotransmission is associated with behavioral and cognitive deficits similar to those found in patients affected by the *Dravet’s* syndrome (78).

Although rare, single mutations of genes encoding for adhesion molecules of the Neuroligin (NL) family found in individuals with autism have been introduced in mice. One of these, the R451C mutation of the *Nlgn3* gene encoding for NL3, has been found in a family with children affected by ASDs (97). NLs are adhesion post-synaptic proteins that, by binding to their presynaptic partners, neurexins, functionally couple the post-synaptic densities with the transmitter release machinery, thus contributing to synapses stabilization (98). Mice carrying the NL3 R451C mutation (NL3^R451C^ knock-in mice) show modifications of GABAergic signaling associated with behavioral deficits reminiscent of those found in autistic children (79, 99). These mice exhibit an increased frequency of mIPSCs in the somatosensory cortex (79) and in the CA3 region of the hippocampus (100) where they contribute to boost GDPs activity. A more detailed analysis of GABAergic microcircuits in the hippocampus has unveiled an increased GABA release at synapses between cholecystokinin (CCK)-positive endocannabinoids-sensitive interneurons and pyramidal cells and a decreased GABA release at synapses between parvalbumin-positive basket cells and principal cells (80). The similar phenotype found at CCK–pyramidal cell synapses in NL3^R451C^ knock in mice and in NL3 KO mice suggests a loss of function consisting in the loss of tonic endocannabinoid signaling at these connections. It is worth noting that NL3^R451C^ knock-in mice present an asymmetric reduction of parvalbumin-positive basket cells across the two hemispheres (82).

Similarly to Földy et al. (80), a reduced probability of GABA release has been found in parvalbumin-positive basket cell–spiny neuron synapses in layer IV somatosensory barrel cortex of juvenile NL3^R451C^ knock in mice. Such deficit determines an alteration of the E/I balance in this cortical layer together with a modification of the temporal window for integration of sensory inputs in principal cells (81). The altered sensory representations may underline deficits in coherent percepts of autistic children.

Mutations in the *En2* gene, coding for the homeobox-containing transcription factor engrailed-2 (EN2), involved in patterning and neuronal differentiation of the midbrain/hindbrain region, have been associated with ASDs. *En2* KO mice have been proposed as a model for ASDs due to their behavioral abnormalities similar to those observed in individuals with ASDs (101). The *En2* gene is also involved in the development or maintenance of GABAergic interneurons as demonstrated by the selective loss of parvalbumin, somatostatin, and neuropeptide Y positive interneurons in the cortex and hippocampus of *En2* KO mice (83). This effect appears to be region-specific since different subpopulations of interneurons are affected in posterior brain areas (102). Deficits in GABAergic neurotransmission account for the higher susceptibility to seizures of En2 KO mice respect to controls (103).

Valproic acid is a histone deacetylase inhibitor, widely used to cure epilepsy and bipolar disorders. VPA is also a potent teratogen since children exposed *in utero* to this drug have a much higher risk of developing an autistic-type behavior than normal children (64). Pups exposed to VPA *in utero* show neuro-developmental abnormalities and behavioral deficits similar to ASDs (104). As in other animal models of ASDs, the autistic phenotype is associated to an altered E/I balance due to a decreased GABAergic signaling, which affects both pre- and post-synaptic sites, leaving the extrasynaptic transmission unaffected (84). Interestingly, like the NL3 model, the VPA model exhibits an asymmetric reduction of parvalbumin-positive cells across the two hemispheres (82). Similarly to *FRM1* KO mice, also in this animal model a loss of oxytocin-mediated GABA_A_-mediated inhibition occurs during the transition from fetal to postnatal life (70). Also here as in Fmr1 KO mice, the depolarizing action of GABA in the hippocampus persists in juvenile life. The excitatory GABAergic drive to principal cells leads to an increased network activity in a broad spectrum of frequencies including gamma oscillations.

The different animal models mentioned here have in common dysfunctions of GABAergic signaling leading to alterations of the E/I balance in selective brain circuits. These alterations can be rescued by selective tools that regulate GABA_A_-mediated synaptic transmission.

## Therapeutic Interventions to Rescue GABAergic Dysfunctions in ASDs

Figure 1 shows a GABAergic synapse with its different constituents.

As summarized in Figure 1A, GABA released from a presynaptic terminal binds to post-synaptic GABA_A_ receptors localized on precise apposition to presynaptic release sites. It binds also to presynaptic and post-synaptic GABA_A_ and GABA_B_ receptors [for a review see Ref. (105)]. Post-synaptic GABA_B_ receptors are localized mainly at perisynaptic sites. Post-synaptic GABA_A_ receptors are maintained in the right place by gephyrin, a scaffold protein which, by interacting with cytoskeletal anchoring elements, contributes to regulate receptor trafficking in and out of the synapses (106). Before being released in a calcium-dependent way, GABA is synthesized by GAD65 and GAD67 and it is stored in presynaptic vesicles by the vesicular transporter VGAT, which uses the electrochemical gradient for H^+^ to shuffle and pack GABA into synaptic vesicles (107). The probability of GABA release is under control of presynaptic receptors including GABA_A_ and GABA_B_ receptors. Adhesion molecules of the neuroligin–neurexin families ensure the cross-talk between post and presynaptic elements of the synapses (98). The figure also shows that the presynaptic terminal contains sodium channels, since, as already mentioned, a selective mutation of these channels in GABAergic interneurons of the forebrain is responsible for the Dravet’s syndrome. After being released, GABA is taken up into nerve terminals and astrocytes by GABA transporters (GATs) localized on presynaptic nerve terminals and astrocytes.

Figure 1B shows the direction of chloride fluxes through post-synaptic GABA_A_ receptors in immature and mature neurons. The influx or efflux of chloride in and out of the cells is mainly dictated by the activity of two developmentally regulated cation–chloride co-transporters, the NKCC1 and KCC2, which pump chloride inside and outside of neurons, respectively (8). Therefore, different components of the synapses can be selectively targeted by agents that can rescue their functions.

Due to the co-morbidity of ASDs with epilepsy, anticonvulsants are widely used for the symptomatic treatment of these disorders (108). Some of these drugs have also an anti-anxiety effect and may indirectly increase GABA_A_ neurotransmission by promoting the synthesis of GABA or by inhibiting their reuptake or breakdown. Interestingly, treatment with vigabatrin, which blocks GABA catabolism by inhibiting GABA transaminase, is able to control seizures and to improve the autistic behavior of children affected by tuberous sclerosis (109).

γ-Aminobutyric acid agonists have proved to be effective in normalizing the E/I imbalance in animal models of Autism: they can either directly enhance inhibition or indirectly reduce excitation. Thus, in BTBR mice [an animal model of idiopathic autism, Ref. (110)] or in *Scn1a*^+/−^ mice [a monogenic model of ASDs, Ref. (78)], in which a reduced GABA_A_-mediated inhibition occurs, the treatment with low non-sedative, non-anxiolytic doses of benzodiazepines, or clonazepam, known to enhance GABAergic signaling *via* allosteric modulation of post-synaptic GABA_A_ receptors, leads to an improvement of social and cognitive deficits. Interestingly, in both BTBR and *Scn1a*^+/−^ mice, the up-regulation of α2 and/or α3 containing GABA_A_ receptors subunits by L-838,417, a selective positive allosteric modulator of these subunits, which does not induce tolerance, is able to mimic the effects of benzodiazepines, thus providing an attractive tool to enhance GABAergic neurotransmission and to improve behavioral deficits in ASDs (110). Consistent with this view, clinical trials using α2/α3 selective positive allosteric modulators of GABA_A_ receptors have been developed with AstraZeneca and the National Institutes of Health (http://clinicaltrials.gov/show/NCT01966679).

Animal models of the Rett syndrome are currently used in translational studies for the preclinical evaluation of new therapeutic trials (111). Genetic studies have provided evidence that neuronal dysfunctions and autistic symptoms can be reversed upon restoration of MeCP2 protein in mice in which the *Mecp2* gene has been silenced using a *lox-Stop-lox* cassette (112, 113), suggesting that in this animal model, the neuronal connectivity is not altered and neurons and glia are not permanently damaged by the *Mecp2* loss. Pharmacological studies have demonstrated amelioration of autistic symptoms with IGF-1 (114) or BDNF (115). However, since most of autistic features of the Rett syndrome can be recapitulated by deleting the *Mecp2* gene in GABA releasing neurons (75), these are expected to be rescued by drugs that enhance GABAergic neurotransmission.

Treatment of Fmr1 KO mice (an animal model of *Fragile X* syndrome), exhibiting an hyper-excitability and a GABAergic dysfunction in the basolateral nucleus of the amygdala, with gaboxadol (THIP), a selective agonist at δ subunits containing perisynaptic or extrasynaptic GABA_A_ receptors, results in an increased GABA_A_-mediated tonic conductance and in beneficial effects on learning deficits and behavioral disturbances linked to this disorder. This supports the hypothesis that tonic inhibition is a putative target for the treatment of *Fragile X* syndrome (65, 116). In addition, consistent with the notion that mGluR are important regulators of protein synthesis, which is translationally repressed by the FMR protein, partial inhibition of mGluR5 or inhibition of excessive glutamate release by the GABA_B_ receptor agonist arbaclofen in *Fmr1* KO mice have led to promising results (117, 118). Although the selective activation of GABA_B_ receptors with arbaclofen has the potential to improve social function and behavior in patients with Fragile X syndrome (119), its use in clinical trials is still under debate (120).

It is worth mentioning that in some children affected by ASDs, allosteric modulators of GABA_A_ receptors such as benzodiazepines have a paradoxical effect, increasing anxiety, and aggression (121). Therefore, restoring low [Cl^−^]_i_ and the inhibitory action of GABA, using the selective blocker of the NKCC1 chloride importer bumetanide, may have beneficial effects. In an elegant study, Tyzio et al. (70) have convincingly demonstrated that, treating pregnant VPA rats and *Fragile X* mice shortly before delivery with bumetanide, suppresses in both animal models the excitatory action of GABA and prevents the autistic-like behavior in off springs. Bumetanide may not only reverse GABA action from the depolarizing to the hyperpolarizing direction, but it may also reduce cell excitability by an ephaptic type of mechanism involving regulation of the cell volume and the extracellular space (122). Animal data validate previous findings by the same group, showing amelioration of autistic symptoms in children treated for 3 months with bumetanide (123, 124). Although these studies should be expanded to larger multicenter trials with more restricted inclusion and exclusion criteria and more extended investigations on the dose/response action of the diuretic as well as persistence of action after drug’s withdrawal, the significant improvement of the autistic behavior associated with a statistically significant amelioration of childhood autism rating scale (CARS) and clinical global impressions (CGI) scores in the absence of clear side effects make this diuretic a very promising drug to cure ASDs. In a parallel study, bumetanide was shown to improve accuracy in facial emotional labeling and to increase brain activation in areas involved in social and emotional perception (125).

## Conclusion

Several lines of evidences suggest that ASDs are neuro-developmental disorders characterized by a clear E/I imbalance in selective neuronal circuits. Such disequilibrium appears to be mainly related to heterogeneous defects of GABAergic signaling in different brain structures. This has paved the way toward the development of new drugs for the cure of these devastating disorders. In particular, several studies have shown that drugs acting on GABAergic synapses are able to rescue behavioral deficits in animal models of autism and to ameliorate at least some of the symptoms observed in ASD patients.

In order to successfully translate therapeutic approaches from animal to humans, it is necessary to develop animal models of ASDs that faithfully trace the behavioral alterations detected in ASD patients. In addition, the effects of drugs should be validated in humans in large scale clinical trials with accurate controls.

Since ASDs are developmental disorders, the early pharmacological intervention, guaranteed by an early diagnosis, is essential. This means that very young children will receive drugs, whose side effects have to be carefully considered. For instance, many drugs acting on GABAergic synapses can generate addiction or give rise to paradoxical reactions, as brain circuits are still immature and GABA may still exert a depolarizing and excitatory action which can be prolonged at late stages of development. Only accurate studies and the use of well suited animal models can help to design pharmacological tools with minimal risks.

## Author Contributions

Giada Cellot and Enrico Cherubini wrote the paper.

## Conflict of Interest Statement

The authors declare that the research was conducted in the absence of any commercial or financial relationships that could be construed as a potential conflict of interest.
